# Concentrations of cadmium and lead, but not zinc, are higher in red fox tissues than in rodents—pollution gradient study in the Małopolska province (Poland)

**DOI:** 10.1007/s11356-018-3951-5

**Published:** 2018-12-28

**Authors:** Joanna Ziętara, Izabela A. Wierzbowska, Joanna Gdula-Argasińska, Agnieszka Gajda, Ryszard Laskowski

**Affiliations:** 1 Institute of Environmental Sciences, Faculty of Biology, Jagiellonian University, Gronostajowa 7, 30-387 Kraków, Poland; 2 Department of Radioligands, Faculty of Pharmacy, Jagiellonian University Medical College, Medyczna 9, 30-688 Kraków, Poland; 3 National Institute for Spatial Policy and Housing, ul. Targowa, 45 03-728 Warszawa, Poland

**Keywords:** Trace metals, Accumulation, Carnivores, Rodents, Risk assessment, Extrapolation factor

## Abstract

The main purpose of our research was to assess the chronic exposure of red foxes to Cd, Pb and Zn. We have determined concentrations of these metals in the kidney, liver and muscle of 36 red foxes hunted between December 2002 and March 2003 in differently polluted areas in southern Poland. Tissue concentrations of Pb and Cd in the red foxes significantly co-varied with concentrations of these elements in the soil, and differed between the tissues. We compared concentrations of Pb, Cd, and Zn in red foxes with two rodent species, *Apodemus flavicollis* and *Myodes glareolus*, trapped simultaneously in the same area. Concentrations of Pb and Cd appeared significantly higher in the red foxes than in the rodents, but the slopes of the relationship between metal concentrations in tissues and in soil were similar in all species. This indicates that extrapolation from results of monitoring studies conducted on rodents to mammalian carnivores is possible but requires applying an extrapolation factor.

**Electronic supplementary material:**

The online version of this article (10.1007/s11356-018-3951-5) contains supplementary material, which is available to authorized users.

## Introduction

The transfer of trace metals to terrestrial mammals depends on a number of factors, e.g., the species, its age, different and highly specialised diets, and seasonal variations of food availability (Hunter et al. 1987). The exposure to chemicals differs among herbivores, omnivores, and carnivores due to specific ways in which contaminants move through food webs (Smith et al. 2007). Also, the type of food is an important factor that determines the availability of metals for assimilation into the organism. For example, zinc is more readily available in meat than in cereal grains. A high-protein diet of carnivores increases the uptake of zinc due to chelating of the metal ions by histidine and cysteine (Sandstead 1988).

Until recently, most of the studies concerning the accumulation and effects of trace metals in wild terrestrial mammals were carried out almost exclusively on rodents. The use of rodents for risk assessment is a commonly accepted practise (Talmage and Walton 1991; Sawicka-Kapusta 1994, 1999; Sheffield et al. 2001; Gdula-Argasińska et al. 2004, 2005). In contrast, similar studies on top terrestrial carnivores are scarce, and only recently, an increasing interest in this topic can be observed (Millán et al. 2008; Kalisinska et al. 2012; Naccari et al. 2013; Binkowski et al. 2016; Lazarus et al. 2017; Rodríguez-Jorquera et al. 2017). The lack of data on large carnivores stems from the fact that in most European countries, they are protected by the Habitats Directive (Council Directive 92/43/EEC 1992) and Berne Convention (1979), and the availability of their tissues for studies is limited to two species, the red fox and golden jackal, whose populations are increasing (Arnold et al. 2012) and the regulation of their populations by hunting is allowed in most of Europe. This opens the possibility to assess metal transfer to large carnivores by analysing metal concentrations in their tissues and their prey. Rodents constitute the main volume of the red fox prey, making them the most important pathway for transfer of pollutants from the environment along the food chain. In our study, we focused on comparing concentrations of the most important metal pollutants, namely cadmium (Cd), lead (Pb), and zinc (Zn), in tissues of red foxes with concentrations of these metals found in the same tissues of two rodent species, *Apodemus flavicollis* and *Myodes glareolus*. This allowed us to assess the transfer of metal pollutants along this short trophic chain and to find out if it is possible to extrapolate data on metal concentrations in rodent tissues to red foxes.

Like in many European countries, the red fox (*Vulpes vulpes*) population in Poland has increased substantially in recent years due to an oral vaccination programme against rabies which began in 1993. The following years showed an increased number of hunted foxes in Poland. According to the Polish Hunting Association (http://www.czempin.pzlow.pl), the mean annual harvest of this species in Poland between 2007 and 2017 was 137,470 including 5400 from the Małopolska province alone, where the study was performed. The density of red fox population in Małopolska (2002/2003) was about 5 individuals/10 km^2^. The mean harvesting in this area was 5398 individuals (https://www.pzlow.pl/index.php/statystyki-lowieckie/385-odstrzal-wazniejszych-zwierzat-lownych-w-sezonie-2002-2003-z-dnia-31-03-2003). The red fox population in Poland is currently estimated at 189,000 individuals. These numbers indicate that the hunting pressure in Poland on red fox population is high, reaching about 70% of the estimated annual total population number. At the same time, the red fox is one of the most widely distributed and common predators worldwide (Kidawa and Kowalczyk 2011). These factors make this species a prospective candidate for monitoring programmes, including studies on the accumulation of pollutants in top terrestrial carnivores and pollution effects on their populations.

The major problem with metals as pollutants is their persistence in the environment; contamination of the environment with metals may thus decrease habitat quality for a long time. In highly industrialised and urbanised areas, such as most of Europe, the preservation of high quality habitats may appear more important for the conservation of wildlife than the increase of protected areas (Klok et al. 2000). The availability of data on potential risks arising from environmental pollution can facilitate the efforts directed at the conservation of endangered species. Our study differs from earlier ecotoxicological field studies on red fox populations (Kalisińska et al. 2012; Naccari et al. 2013; Binkowski et al. 2016) by the fact that we collected specimens from a large area, representing a broad range of metal contamination of soils (whole Małopolska province—15,182.87 km^2^, southern Poland). Zn, Cd, and Pb concentrations were determined in the liver and kidney that are the main target organs of these metals in an organism. We also analysed the muscle tissue. The results were compared with data originating from the study by Gdula-Argasińska et al. (2005) from the same area on metal concentrations in two rodent species. This enabled us to study the correlations between metal concentrations in the red fox tissues and in their natural habitat and tissues of the main prey of the red fox. We also discuss the possibility for interspecies extrapolations based on monitoring programmes on rodents.

The study aimed at answering the following specific questions: (1) Do the concentrations of trace metals in the tissues of the red fox depend on the metal level in the soil? (2) Do sex and age affect the accumulation of the metals in the tissues? (3) Do the tissues differ in metal concentrations? (4) Can the data on metal concentrations in rodents be used to predict concentrations of the metals in red foxes?

## Materials and methods

**Fig. 1:**
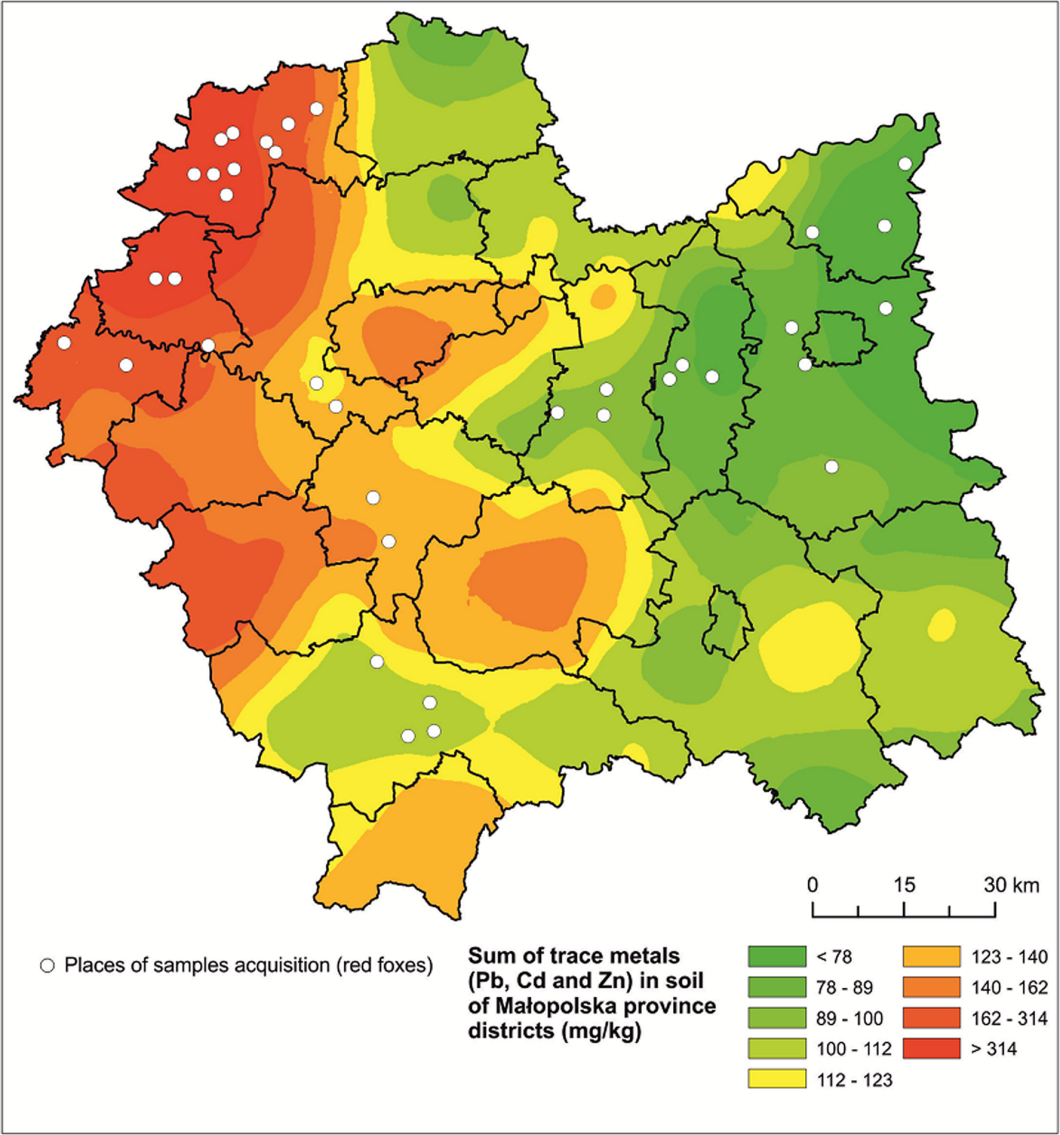
The map of the Małopolska province with places (white dots) where red foxes were harvested and the sum of concentrations (mg/kg soil d.w.) of three trace metals (Pb, Cd, and Zn) (after Tokarz and Turzański, 1999)

The Małopolska province located in southern Poland is one of the largest industrial and urbanised regions in the country. It comprises both pristine areas (the eastern part) and areas highly polluted with trace metals (the north-western part). According to Tokarz and Turzański (1999), in the areas covered by our study, the concentration of cadmium in the soil ranges from 0.01 to 33.0 mg/kg, of lead from 3.6 to 2787 mg/kg, and of zinc from 2.4 to 5754 mg/kg dry weight (d.w.). The data on metal concentrations in the soil used in our study were provided as average concentrations for each county of the Małopolska province (mean county size 806.5 km^2^), based on approximately 100 soil samples analysed in each county (Tokarz and Turzański 1999). From this area, 100 foxes were obtained from hunters during the 2002/2003 hunting season and tested for the presence of the rabies virus by the Regional Veterinary Inspectorate in Kraków. From the foxes that were not virus carriers, liver, kidneys, and femora with a fragment of the muscle were collected for further studies. Individual tissues were transported in plastic bags and stored frozen at – 20 °C till analysis. In order to determine the age of individuals, we removed and examined the lower left canine. From among all the collected samples, we selected 36 individuals originating from the areas representing the widest possible range of metal concentrations in the soil (Fig. 1).

In the study on rodents from the same area (Gdula-Argasinska et al. 2005), in each site, yellow-necked mice (*Apodemus flavicollis*, Melchior 1834) and bank voles (*Myodes glareolus*, Schreber 1780) were captured using life and snap traps. All animals caught were sexed and aged by weighing the eye lens. Metal concentrations in the livers, kidneys, and spleen of the rodents were investigated. The study was approved by the Local Ethics Commission. For further details see Gdula-Argasinska et al. (2005).

### Chemical analyses

The concentrations of Pb, Cd, and Zn were determined in the muscles, livers, and kidneys using Atomic Absorption Spectrometry (AAnalyst 800, Perkin-Elmer). The flame technique was used for Zn determination and the graphite-furnace method for Cd and Pb.

Prior to use, all laboratory vessels for tissue preparation and mineralisation were acid-cleaned in a bath of 10% (*v*/*v*) nitric acid, then rinsed with ultrapure deionised water (Labconco Water PRO PS) and oven-dried. Tissues and organs, after initial drying in an oven at 60 °C to constant weight, were homogenised using an agate mill. Then, the samples of approximately 0.1 g of homogenised tissues were transferred to 10-ml glass vials, dried at 105 °C, and weighted to the nearest 0.001 g. Finally, samples were digested in an ultrapure mixture of nitric (Suprapur, Merck, Germany) and perchloric acids (70%, Merck, Germany) in the ratio 7:1 (*v*:*v*) using a thermoblock.

After complete digestion and evaporating excess of the acids, the solutions were diluted with deionised water to a final volume of 10 ml and transferred to polyethylene flasks. The reagent blanks were used during each mineralisation procedure. During the analyses, every ten samples blank and control samples were analysed to assure the proper quality of the measurements. Each sample was analysed twice.

Limits of analytical method were established and the accuracy of the whole procedure was checked with the certified reference material (bovin liver 185r, PROMOCHEM) analysed in three replications. The percentage recovery was between 91.5 and 136.5% for Cd, 112.5 and 128.4% for Pb, and 92.3 and 100.01% for Zn. The detection limit for Cd, Pb, and Zn was 0.016, 0.408, and 0.06 μg/l, respectively, and it was calculated as three standard deviations for the mean of blanks measurement. Concentrations lower than the limit of detection were only noted in two Pb samples and they were replaced with the value of half mean from blanks. All concentrations of trace metals are given on a dry weight (d.w.) basis.

### Age determination

The age of individuals was estimated using the left lower canine tooth (C1) according to Goszczyński (1995), based on the root canal width. We used the regression equation (Eq. 1) for the relationship between the canine root canal width and the age of an individual from Kozłowski (2000),

1 $$ \mathrm{Y}=75.587-2.87645\ast \mathrm{X} $$

where *Y* is the ratio of root canal width to the width of the whole tooth (%) and *X* is the age of an individual in months.

### Calculations and statistics

To better illustrate the contamination level across the study area and to see to what extent the increase in soil pollution is reflected in different tissues of red foxes, we calculated the concentration increase factor (CIF) as the ratio in metal concentrations between highly polluted and low-polluted areas for soil and red fox tissues (Eq. 2).

2 $$ \mathrm{CIF}=\mathrm{concentration}\ \left(\mathrm{highly}\ \mathrm{polluted}\ \mathrm{areas}/\mathrm{low}\ \mathrm{polluted}\ \mathrm{areas}\right)\ \mathrm{based}\ \mathrm{on}\ \mathrm{mean}\ \mathrm{and}\ \mathrm{median}\ \mathrm{metal}\ \mathrm{concentrations} $$

To compare metal accumulation in females and males, we calculated females:males ratio (F/M) for metal concentrations in all analysed tissues (Eq. 3).

3 $$ \mathrm{F}/\mathrm{M}\ \mathrm{ratio}=\left(\mathrm{females}/\mathrm{males}\right)\ \mathrm{tissue}\ \mathrm{concentration} $$

This could be done, however, only for the foxes originating from the highly contaminated area because only there the number of females and males was similar (8 and 6, respectively) and sufficient to offer meaningful results. Additionally, the kidney:liver ratio was calculated for all individuals from each of the three differently polluted (low, medium, and highly) areas. All factors were calculated based on both the average and the median values because of the high variability and highly skewed distributions of metal concentrations in tissues.

Normality of distributions was tested using the Chi-square and Kolmogorov-Smirnov tests (Zar 1999). If the data distribution deviated significantly (*p* ≤ 0.5) from the normal distribution, the data were log-transformed. After the transformation, all data followed the normal distribution. Differences in metal concentrations between tissues and sexes were tested for significance with two-way analysis of variance (ANOVA) with metal concentration in the soil and age of individuals as covariates. If the significant effect was found, means were separated with Tukey’s test (Zar 1999).

The relationships between metal concentrations in the soil and fox tissues were analysed with regression analysis. The slopes and intercepts of the regressions for the liver, kidneys, and muscles were compared with the dummy variable method. The same method was used to compare relationships between Pb, Zn, and Cd concentrations in the soil and selected organs (livers or kidneys) of foxes and two rodent species: the yellow-necked mouse and the bank vole. The data for the rodents were taken from the study by Gdula-Argasińska et al. (2005) that was performed in the same area (Małopolska province); the animals were trapped at the same time as the foxes, i.e., between autumn 2002 and spring 2003. Because the analysis showed that regression lines for all three species have the same slopes but differ in intercepts, the differences in tissue concentrations between the species were analysed with ANOVA with metal concentration in the soil as a covariate; means were separated with Tukey’s test. All statistical analyses were made using Statgraphics Centurion XVI.I (StatPoint Technology, Inc.).

## Results

### Concentrations of Pb, Cd, and Zn in the tissues of the red fox

The age was estimated for only 28 individuals out of the total 36 used for the trace metal analysis because of missing teeth. The age ranged from 3 to 22 months. Most of the individuals were between 11 and 15 months old with the median 14.5 months.

The concentration of Pb in the studied organs varied widely (Table 1). In the liver, where the concentration of lead reached the highest values as compared to other organs, the range was 0.08 to 178.01 mg/kg. Lead concentrations in the liver and kidney were significantly higher than in the muscle (*p* < 1 × 10^−5^, Fig. A1). No correlation was found between the age of red foxes and Pb concentration in tissues (*p* = 0.38). The highest Pb concentration in the liver, 178.01 mg/kg (this value was identified as outlier and withdrawn from further analysis), was found in a 13-month-old male from Tarnów (a city in the eastern part of the Małopolska province). The next highest Pb concentration (23.7 mg/kg) was found in a female from the same area (no age data due to missing teeth). The male also had a high kidney Pb concentration (47.23 mg/kg), with the median for the whole group being 0.78 mg/kg. The concentrations of Pb in the liver of both individuals were above 10 mg/kg-the level assumed to be toxic (Ma 1996). Pb concentration was significantly higher in females than in males (*p* = 0.02, Fig. A2). Regression analysis revealed a significant relationship between Pb concentrations in the soil and in fox tissues (*p* < 1 × 10^−5^), with no difference in slopes (*p* = 0.62) and significant differences in intercepts (*p* = 1 × 10^−4^) between tissues.

**Table 1:** Concentrations of trace metals (Pb, Cd, and Zn) in the tissues of red foxes from the Małopolska province (mg/kg d.w.)

Metal	Organ	*N*	Mean	Median	Standard deviation	Range
Pb	Muscle	36	0.40	0.29	0.42	0.02–1.99
	Liver	36	7.43	0.82	29.77	0.08–178.01
	Kidney	36	2.26	0.78	7.77	0.07–47.23
Cd	Muscle	36	0.81	0.04	3.20	0.01–18.96
	Liver	36	7.46	1.40	15.06	0.1–70.07
	Kidney	36	22.93	5.09	57.86	0.25–325.55
Zn	Muscle	36	89.50	81.26	30.46	53.11–193.74
	Liver	36	130.64	130.13	27.62	66.07–189.84
	Kidney	36	73.73	72.16	11.23	57.55–108.84

The analysis of Cd concentrations in tissues revealed one extreme measurement. The individual originating from Olkusz (the industrialised western part of the Małopolska province) contained 18.96 mg/kg of this metal in the muscles. The two-way ANOVA showed a significant effect of sex (*p* = 0.035) and tissue (*p* < 1 × 10^−5^) and significant covariance of Cd concentrations in tissues with soil Cd concentration (*p* = 0.021). Average Cd concentration was significantly higher in the kidney and liver compared to the muscle, with no significant differences between the kidneys and liver (Fig. A3). Females had higher Cd concentrations than males (*p* < 0.0035, Fig. A4). The linear model relating Cd concentrations in fox tissues to Cd concentration in soil was significant at (*p* < 1 × 10^−4^), with significant differences between tissues in intercepts (*p* < 1 × 10^−4^) but no difference in slopes (*p* = 0.73).

There was no relationship between Zn concentration in the tissues and age, sex or Zn concentration in the soil. Average Zn concentration was highest in the liver (Fig. A5).

### Comparison of Cd, Pb, and Zn accumulation in the red fox (*Vulpes vulpes*) and in two rodent species: the yellow-necked mouse (*Apodemus flavicollis*) and bank vole *(Myodes glareolus*)

**Fig. 2:**
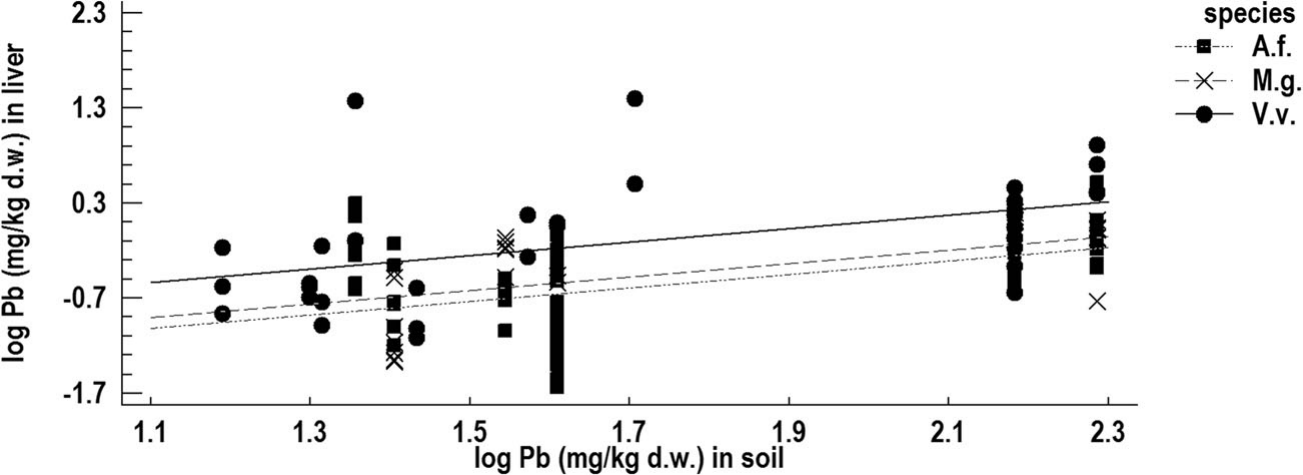
The relationship between Pb concentration in the soil and in the liver of the red fox (V.v.), yellow-necked mouse (A.f.), and bank vole (M.g.) from the Małopolska province

The relationship between Pb concentrations in the soil and in the livers of red foxes and the two rodent species were highly significant (*p* < 1 × 10^−5^). Comparison of the regression lines for the foxes and the rodents showed a significant difference in intercepts (*p* < 1 × 10^−5^, Fig. 2) and no difference in slopes (*p* = 0.447).

The equal slopes mean that the rate of increase of Pb in tissues with increasing Pb concentration in the soil is the same in all three species. The differences in intercepts indicate, in turn, inherently different Pb concentrations in the livers of the three species. ANOVA with Pb concentration in the soil as a covariate confirmed highly significant differences (*p* < 1 × 10^−5^) between species in mean Pb concentrations in the liver. The post-hoc Tukey test showed that the red fox had significantly higher Pb concentration in the liver compared to the two rodent species, while the rodents did not differ from each other (Fig. A6).

**Fig. 3:**
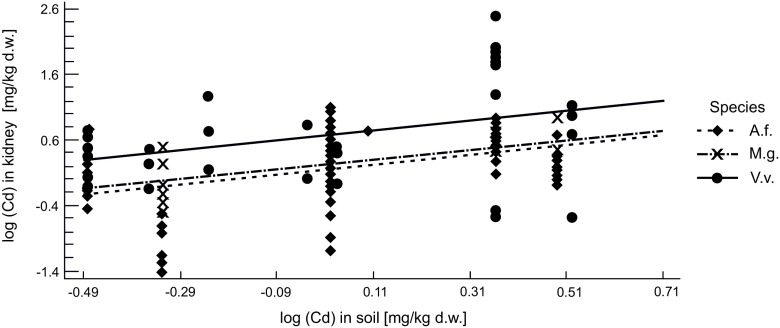
The relationship between Cd concentration in the soil and in the kidney of the red fox (V.v.), yellow-necked mouse (A.f.), and bank vole (M.g.) from the Małopolska province

Similar to Pb, the relationship between the Cd concentration in the soil and in the kidneys of the red foxes and the rodents was also highly significant (*p* < 1 × 10^−5^), with significant differences between species in intercepts (*p* < 1 × 10^−4^) and no difference in slopes (*p* > 0.9, Fig. 3).

The equality of the slopes allowed us to perform ANOVA to check the differences in average kidney Cd concentration between the three species. The ANOVA confirmed highly significant differences between the species (*p* < 1 × 10^−4^), with Tukey’s test showing that Cd concentration in the kidneys of the red foxes was higher than in the rodent species, while the two rodent species did not differ from each other (Fig. A7).

**Fig. 4:**
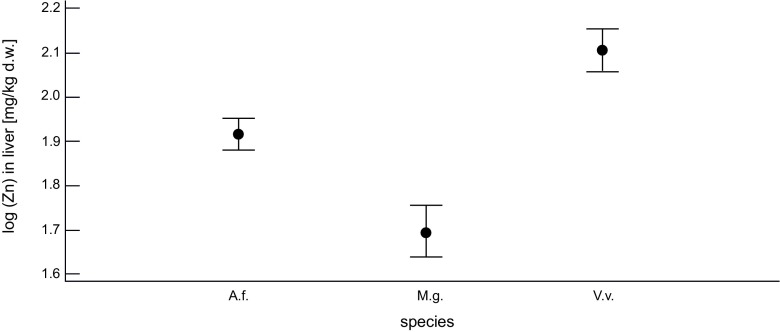
The concentration of Zn in the livers of the red fox (V.v.), yellow-necked mouse (A.f.), and bank vole (M.g.) from the Małopolska province. Means and 95% Tukey HSD intervals

In contrast to Pb and Cd, the relationship between Zn concentration in the soil and in the livers of all three species was nonsignificant (*p* = 0.53). However, ANOVA showed highly significant differences between Zn concentration in the liver of the species (*p* < 1 × 10^−4^). The Tukey’s test showed that all the tested species differed from each other, with the highest Zn concentration in the red fox, and the lowest in the bank vole (Fig. 4).

Because most earliest studies on metal concentrations in tissues of top predators did not cover such a broad range of metal pollution as our study, for the sake of comparing our data against other published studies, we combined the data from Małopolska into three zones representing low, medium, and high metal pollution in soil (Table 2). Accordingly, data on metal concentrations in red fox tissues were also combined into the three pollution zones (Table 3). The calculated concentration increase factors (CIF), females:males (F/M) ratios for all analysed tissues, and kidney:liver ratios are presented in Table 4.

**Table 2:** Mean and median concentrations (mg/kg d.w.) of Cd, Pb, and Zn in the soil of differently polluted areas (L, low; M, middle; and H, high) in Małopolska province (Poland)

	Cd			Pb			Zn		
	Mean	SD	Median	Mean	SD	Median	Mean	SD	Median
Soil (L)	0.43	0.11	0.44	19.34	2.88	19.88	64.92	9.80	59.54
Soil (M)	1.05	0.12	1.04	37.67	9.35	37.36	93.59	15.46	88.01
Soil (H)	2.57	0.46	2.29	163.88	19.04	152.28	300.0	113.32	230.94

**Table 3:** Mean and median concentrations (mg/kg d.w.) of Cd, Pb, and Zn in the tissues of red foxes collected in the differently polluted areas of the Małopolska province: L, low pollution; M, medium pollution; H,high pollution

	Cd			Pb			Zn		
Tissue	Mean	SD	Median	Mean	SD	Median	Mean	SD	Median
Liver (L)	1.48	1.71	0.96	2.29	6.75	0.26	121.93	18.22	119.21
Liver (M)	1.73	1.60	1.00	1.01	1.05	0.84	123.35	13.39	130.09
Liver (H)	16.8	21.27	3.63	2.21	2.16	1.60	143.42	36.72	146.02
Kidney (L)	3.92	4.85	2.80	0.47	0.65	0.215	69.45	7.23	71.38
Kidney (M)	4.90	3.81	5.62	0.73	0.54	0.89	69.49	5.93	69.39
Kidney (H)	52.18	86.46	16.36	1.56	0.95	1.59	80.41	13.81	77.96
Muscle (L)	0.14	0.28	0.03	0.21	0.13	0.17	80.62	9.01	80.16
Muscle (M)	0.05	0.035	0.04	0.36	0.32	0.29	102.24	45.26	83.13
Muscle (H)	1.91	5.03	0.05	0.60	0.57	0.44	89.57	31.17	83.15

**Table 4:** Factors calculated based on metal concentrations in the soil and tissues of red foxes coming from three differently polluted areas of the Małopolska province: L, low pollution; M, medium pollution; H, high pollution

	Cd		Pb		Zn	
	Mean	Median	Mean	Median	Mean	Median
Soil CIF^a^	5.93	5.2	8.47	7.66	4.62	3.88
Liver CIF	11.36	3.78	0.96	6.09	1.18	1.22
Kidney CIF	13.3	5.84	3.13	7.4	1.16	1.09
Muscle CIF	13.5	1.74	2.98	2.85	1.11	1.04
Liver F/M ratio (H)^b^	2.54	10.19	1.12	2.70	0.98	0.98
Kidney F/M ratio (H)	3.79	7.41	2.05	2.39	1.21	1.23
Muscle F/M ratio (H)	33.77	15.30	3.25	2.30	0.82	0.97
Kidney:Liver ratio (L)	2.65	2.91	0.26	0.81	0.57	0.60
Kidney:Liver ratio (M)	2.82	5.65	0.73	1.05	0.56	0.53
Kidney:Liver ratio (H)	3.11	4.5	0.70	0.99	0.56	0.53

^a^*CIF* concentration increase factor (highly polluted vs. low polluted areas) calculated based on mean and median metal concentrations

^b^F/M ratio (H): females (*n* = 8) vs. males (*n* = 6) from highly polluted area

When comparing median concentrations of Cd, Pb, and Zn in soils from areas with the lowest and highest pollution level in Małopolska province, and also in the tissues (liver, kidney, and muscles) of red foxes coming from those areas (Table 3), we noted that the fivefold increase in Cd concentration in soil (CIF_median_ = 5.2) corresponded to approximately fourfold concentration increase in livers (CIF_median_ = 3.78), almost sixfold in the kidney (CIF_median_ = 5.84), and twofold in the muscles (CIF_median_ = 1.74). However, the presence of a few individuals with extremely high Cd concentrations in tissues skewed the distribution of Cd concentrations; CIFs based on mean concentrations exceeded 13 for the kidney and muscle. In the highly polluted area, females had higher Cd concentration than males in all analysed tissues: ten times more in the liver, seven times more in the kidney, and 15 times more in muscles for median Cd concentration. The ratio kidney:liver for Cd was the lowest in the least polluted area and the highest in the medium-polluted (Table 4).

The almost eightfold increase in Pb concentration in soil (CIF_median_ = 7.66) corresponded to sixfold increase in the liver (CIF_median_ = 6.09), almost sevenfold in the kidney (CIF_median_ = 6.74), and threefold in the muscles (CIF_median_ = 2.85). In the most polluted areas, females also had more elevated Pb concentrations in the tissues than males (Table 4). The median kidney:liver ratio for Pb was lowest in the area with the lowest pollution level and highest in the medium-polluted area, but generally, the differences between the sites of different pollution level were much smaller than for Cd: L = 0.81, M = 1.05, and H = 0.99 (Table 4).

The approximately fourfold increase in Zn concentration in soil (CIF_median_ = 3.88) corresponded to negligible increase in the livers (1.2 times) and kidney (1.1 times), and no increase in muscles (CIF_median_ = 1.0). In the most polluted areas, no differences between females and males in the median Zn concentration in the liver and muscles were found. However, females had slightly higher concentrations (F/M ratio = 1.2) of this metal in the kidneys. The median Zn kidney:liver ratio was negligibly higher in the least polluted area (L = 0.6, M = 0.53, and H = 0.53) compared to the remaining areas.

## Discussion

The median liver and kidney concentrations of Pb in foxes from Małopolska increased 6.1 and 7.4-fold respectively between the least and most polluted areas (Table 4). We noted, however, extremely high Pb concentrations in two individuals coming from the least polluted area: a male and a female from the suburbs of Tarnów city. The liver Pb concentration in the male (178 μg/g d.w.) and in the female (23.7 μg/g d.w.) exceeded the level considered to be toxic, 10 mg/kg d.w. (Ma 1996). The concentration of Pb in the kidney in the same male (47.23 μg/g d.w.) was also above the concentration considered as toxic (25 mg/kg d.w.; Ma 1996). Such high concentrations could result from pellets containing lead; however, the buckshot was not found during necropsy. Another possibility is that foxes ate the carrion of game animals shot with Pb bullets which are still in use in Poland. The fact that both individuals came from the same place and were harvested in the vicinity of an urbanised area may indicate lead contamination resulting from feeding in the city. Cities are known for being notoriously contaminated with Pb, resulting from the past traffic pollution as a consequence of the previous use of leaded petrol. Once released to the environment, metals can remain in the soil for a very long time. For example, the estimated 10% residence time in the contaminated soil (i.e., the time needed for a concentration to decrease by 10%) is 10–20 years for Zn and Cd, about 100 years for Cu, and more than 200 years for Pb (Tyler 1978).

The comparison of Cd, Pb, and Zn concentration (mg/kg) d.w. in red foxes from Małopolska and different studies in Europe is presented in Table 5. The comparison of median Pb concentrations in tissues of the foxes from Małopolska with data obtained from foxes from different distances from the agglomeration of Zurich (Dip et al. 2001) showed that the foxes from Małopolska contained, on average, less lead. This can be explained by the fact that a greater number of individuals in the study of Dip et al. (2001) were from urban and suburban areas (a zone of 0.5 km around Zurich), with significantly higher levels of Pb than in rural foxes. Jankovska et al. (2010) found the median liver Pb concentration ranging from 0.26 to 0.57 μg/g d.w. in the foxes coming from the area influenced by pollutants from petrochemical industries and brown coal power plants in the Czech Republic. These concentrations were within the concentration range found in our foxes from Małopolska’s least and moderately polluted areas: 0.26 and 0.84 μg/g d.w. respectively (Table 3). The mean Pb liver concentration in red foxes from central Italy (Corsolini et al., 1999) and north-western Spain (Perez-Lopez et al. 2015) was lower (0.89 and 0.81 μg/g d.w. respectively) than mean concentrations in foxes from Małopolska’s low, moderately, and highly polluted areas (2.29, 1.01, 2.21 μg/g d.w. respectively). In south-western Poland (Binkowski et al. 2016), Hungary (Heltai and Markov 2012) and Romania (Farkas et al. 2017), the authors found the following concentrations of Pb in the livers of red foxes: 1.66, 1.68, and 1.88 μg/g d.w. respectively. All these concentrations fall within the levels found in the foxes from Małopolska province.

**Table 5:** The comparison of Cd, Pb, and Zn concentration (mg/kg d.w.) in red foxes from Małopolska and different studies in Europe

Liver			Kidney			Muscles			Location	References
Cd	Pb	Zn	Cd	Pb	Zn	Cd	Pb	Zn		
7.46 ± 15.06 (1.40)	7.43 ± 29.77 (0.82)	130.64 ± 27.62 (130.14)	22.93 ± 57.86 (5.09)	2.26 ± 7.77 (0.78)	73.73 ± 11.23 (72.16)	0.81 ± 3.2 (0.04)	0.4 ± 0.42 (0.29)	89.5 ± 30.46 (81.26)	Poland (Małopolska)	This study
1.73 ± 1.7 (1.07)	4.0 ± 12.03 (1.93)	149.52 ± 55.66 (139.32)	6.59 ± 6.5 (4.41)	2.59 ± 4.09 (1.68)	96.36 ± 35.45 (91.81)	n.d.	n.d.	n.d.	Switzerland	Dip et al. (2001)^a^
(0.54)	(0.42)	(102.9)	(2.03)	(0.53)	(72.63)	n.d.	n.d.	n.d.	Czech Republic	Jankovska et al. (2010)^b^
0.6 ± 0.52	0.89 ± 0.69	n.d.	n.d.	n.d.	n.d.	n.d.	n.d.	n.d.	Central Italy	Corsolini et al. (1999)
0.58 ± 0.39 (0.61)	0.81 ± 0.74 (0.63)	77.0 ± 25.0 (78.0)	1.28 ± 1.26 (0.92)	0.06 ± 0.05 (0.03)	17.0 ± 7.0 (17.0)	n.d.	n.d.	n.d.	North-western Spain	Perez-Lopez et al. (2015)
1.29 ± 1.22	1.66 ± 3.07	128.26 ± 35.35	1.66 ± 2.89	1.64 ± 1.72	58.58 ± 28.88	2.66 ± 0.85	0.64 ± 0.94	87.31 ± 35.53	South-western Poland	Binkowski et al. (2016)
0.49 ± 0.14	1.68 ± 0.38	156.93 ± 44.77	0.82 ± 0.6	2.63 ± 1.23	87.16 ± 24.85	n.d.	n.d.	n.d.	Hungary	Heltai and Markov (2012)
3.51 ± 6.82	1.88 ± 3.43	n.d.	n.d.	n.d.	n.d.	n.d.	n.d.	n.d.	Romania	Farkas et al. (2017)

The values are presented as mean ± SD (median) and given in milligrams per kilograms d.w.

^a^The values are calculated from data on wet weight basis (assuming the tissue water content = 70%)

^b^The values are presented as mean from metals concentration in parasitized and non-parasitized foxes

The kidney median Pb concentration in foxes from the Czech Republic ranged from 0.26 to 0.88 μg/g d.w. and corresponded to the median concentrations found in foxes from the low and moderately polluted areas in Małopolska (0.215 and 0.89 μg/g d.w. respectively (Table 3). When comparing mean Pb kidney concentration, our foxes had in general lower Pb levels (0.47, 0.73, and 1.56 μg/g d.w. for low, moderately, and highly polluted areas respectively), except for one individual found in the suburbs of a city (47.23 μg/g d.w.), than red foxes from Hungry (Heltai and Markov 2012), Switzerland (Dip et al. 2001) and south-western Poland (Binkowski et al. 2016) with respective values 2.63, 2.59, and 1.64 μg/g d.w. The red foxes from north-western Spain (Perez-Lopez et al. 2015) had the lowest kidney Pb concentration (0.06 μg/g d.w.) ever recorded in Europe.

The Zn, Cd, and Pb concentrations in the red foxes muscles were determined only in our, Binkowski’s et al. (2016) and Naccari’s et al. (2013), studies. The mean Pb concentration in muscles of our foxes from the most polluted area (0.6 μg/g d.w.) was similar to the foxes analysed by Binkowski et al. (2016) from the relatively unpolluted area in south-western Poland (0.64 μg/g d.w.). Polish red foxes from both studies had higher mean Pb muscle concentrations than foxes from Sicily (0.04 μg/g d.w.) studied by Naccari et al. (2013). The similarity of Pb concentrations in foxes from both Polish populations suggests that lead contamination is not necessarily related exclusively to industrial pollution but may originate from local sources and, perhaps, past traffic when soil was polluted by gasoline-born Pb.

We did not find significant correlation between the concentration of Pb in various organs and the age of red foxes. Dip et al. (2001) reported a temporary increase in the concentration of Pb in the liver of foxes: the concentration increased in juveniles under 13 months of age then decreased progressively with age, especially in animals over 24 months. The pattern was observed only in individuals inhabiting an urbanised area. The increase with age in the concentration of Pb, Cd, and Hg in tissues of red foxes was found by Millán et al. (2008), who analysed different carnivore species from Spain. In red foxes from Małopolska, individuals from urban areas accounted for a small percentage of the total sample. Furthermore, in our study, there was no single individual over 24 months of age. According to Dip et al. (2001), a transient increase in the concentration of Pb in the liver of red foxes is probably due to increased absorption of this trace metal as a result of an increase in the absorption of calcium during the skeletal growth of juveniles, while the subsequent decrease of Pb concentration in this organ is likely due to the elimination or slow build-up of this metal into the bones.

Lead concentration in female red foxes from Małopolska was significantly higher than in males. However, the number of individuals of both sexes differed (23 males and 13 females), and in the areas with low, medium, and high metal pollution, the ratio females:males was 2:11, 3:6, and 8:6, respectively. For this reason, we additionally made the comparison for only the highly polluted areas, where both sexes were more equally represented. Compared to the males, females in these areas had 2.7 times higher median Pb concentration in their livers, 2.4 times higher concentration in kidneys, and 2.3 times higher in muscles (Table 4). Similar gender differences were found by Millán et al. (2008) in Pb liver concentration of red foxes from Spain and by Naccari et al. (2013) in Pb skin concentration of red foxes from Sicily. Perez-Lopez et al. (2015), in the analysis of metal concentrations in the kidney and liver of a large group of red foxes (110 females and 140 males) from rural areas in north-western Spain, did not find any significant differences between genders in Cd, Pb, and Zn concentrations. Also, Dip et al. (2001) did not report such differences, but they refer to other authors (Ansorge et al. 1993; Corsolini et al. 1999) who found similar differences between sexes in the red fox. The lack of differences between sexes in trace metals accumulation in red foxes studied by Perez-Lopez et al. (2015) may result from the fact that their study was carried in the relatively unpolluted area. At low exposure level, the differences between genders may remain nonapparent. However, Naccari et al. (2013) found such differences also in a relatively unpolluted area in Sicily.

The differences in the accumulation of trace metals between the sexes can be explained by the fact that metallothioneins, the metal binding proteins, show some gender-specific differences (Burger et al. 2007, Nordberg et al. 1998). In turn, the reproductive status, such as pregnancy or lactation, which is associated with increased energy demand and, hence, higher consumption rate, may result in higher transfer of metals to female bodies. During pregnancy and reproduction also, the demand for some essential elements, such as Ca, Cu, Fe, Mg, and Zn, is increased which may result in increased absorption of other trace metals (Vahter et al. 2007). Furthermore, in some species, including the red fox, females can present different food preferences than males (Kidawa and Kowalczyk 2011).

When analysing metal concentrations found in tissues of red foxes in Małopolska, we may conclude that Pb can reach toxic levels, especially in individuals inhabiting areas adjacent to towns, within urban areas, or close to roads with high traffic in the past. This can be directly linked to the increased synurbanisation of red foxes in Poland and other European countries (Bilandžić et al. 2010; Wierzbowska et al. 2011).

The Lowest Observable Adverse Effect Level (LOAEL) for Cd in the kidneys in laboratory rodents has been estimated at 105 mg/kg d.w. (Shore and Douben 1994). The concentration close to this value (102.2 mg/kg d.w.) was registered in a 17-month-old female, and in another, 15-month-old female, a concentration of 325.55 mg/kg d.w. was found, exceeding more than three times the LOAEL. Both young females were harvested in the north-western part of the Małopolska province. This indicates the possibility in the most polluted part of Małopolska of achieving toxic levels of cadmium in the kidneys of individuals at a relatively young age.

In general, Cd concentration in the kidney was higher than in the liver and muscle. The kidney is the main organ accumulating Cd (Nordberg et al. 2007). When its concentration in the kidney reaches the saturation level, the liver becomes the second most important organ in which the concentration gradually increases in the case of further exposure to the metal (Andrews et al. 1989). The comparison of the concentration of cadmium in the liver to its concentration in the kidney enables estimation of the length of exposure to this metal (Talmage and Walton 1991). In our study, when analysing the kidney:liver ratio of median Cd and Pb concentrations for low, moderately, and highly polluted areas (Table 4), we noted a marked increase in the Cd ratio between low and moderately polluted areas and, subsequently, a decrease between moderately and highly polluted areas (ratios 2.91, 5.65, and 4.5 respectively). The Pb kidney:liver ratio showed similar but less pronounced trend (rations 0.81, 1.04, and 0.98 respectively). The decrease in kidney:liver ratio for Cd concentrations between moderately and highly polluted areas indicates that individuals from the most polluted zone started to show signs of kidney overload by Cd. Similar decrease in kidney:liver ratio of Cd in three rodents species coming from different distances from Cu and Cd refinery was noted by Hunter et al. (1987). The median Cd concentrations in the livers of red foxes from Małopolska pollution gradient ranged from 0.96 to 3.63 μg/g d.w. and were substantially higher than in livers of foxes from the Czech Republic (0.31 to 0.94 μg/g d.w.; Jankovska et al. 2010). The results show that red foxes from Małopolska, and particularly individuals inhabiting the most polluted north-western part of the province, are exposed to higher trophic-chain transfer of this metal. The red foxes from Małopolska’s least polluted areas had mean Cd concentration in the livers (1.48 μg/g d.w.) higher than those found in south-western Poland (1.29 μg/g d.w.; Binkowski et al. 2016), while the foxes from the medium polluted area in this study had similar mean Cd liver concentrations to foxes from Switzerland (1.73 μg/g d.w., after conversion of Dip’s data from wet weight to dry weight assuming 70% water content in the liver). The red foxes from the most polluted area in Małopolska province represent the highest level of Cd in red fox livers ever recorded (mean = 16.8 μg/g d.w., median = 3.63 μg/g d.w.). The lowest known mean Cd liver concentration was found in foxes from Hungary (0.49 μg/g d.w.; Heltai and Markov 2012), followed by foxes from north-western Spain (0.58 μg/g d.w.; Perez-Lopez et al. 2015) and Italy (0.6 μg/g d.w.; Corsolini et al., 1999).

The median Cd kidney concentrations in the foxes from the most polluted part of Małpolska province (16.36 μg/g d.w.) was substantially higher than the highest concentration recorded in Czech Republic (2.96 μg/g d.w.; Jankovska et al. 2010). The highest median value noted by Jankovska et al. (2010) corresponded to the median concentration found in our foxes from the least polluted area. The lowest ever recorded mean Cd kidney concentration was found in Hungarian foxes (0.82 μg/g d.w.; Heltai and Markov 2012), followed by foxes from north-western Spain (1.28 μg/g d.w.; Perez-Lopez et al. 2015) and south-western Poland (1.66 μg/g d.w.; Binkowski et al. 2016). Mean Cd kidney concentration in foxes from Switzerland (6.59 μg/g d.w.; Dip et al. 2001) falls within the range found in foxes from Małopolska (4.9 to 52.18 μg/g d.w.). Foxes from the most polluted areas in Małopolska had the highest level of this metal in their kidneys among all European populations of foxes studied. In contrast, the mean Cd concentration in the red foxes muscles from south-western Poland (2.66 μg/g d.w.; Binkowski et al. 2016) was higher than the mean for foxes from Małopolska, even those inhabiting the most polluted areas (1.91 μg/g d.w.). It is difficult to explain why foxes from south-western Poland had such a high level of Cd in their muscles having at the same time relatively moderate concentration of this metal in their livers and kidneys.

Similar to Pb, Cd concentration was higher in females. When comparing only females and males from the highly polluted areas, the females show 10.2-fold higher median Cd concentration in the livers and 7.4-fold higher concentration in the kidneys and 15.3-fold higher in the muscles. In an extensive study on four top European mammalian predators, Lazarus et al. (2017) found that bears and wolf females also showed generally higher metal concentrations than males. The difference between the sexes may be due to the reasons suggested already above for lead.

Also similar to Pb, Cd concentrations showed no correlation with the age of red foxes. This is inconsistent with the findings of other authors investigating Cd concentration in various mammalian species (Komarnicki 2000; Dip et al. 2001; Parker and Hamr 2001). Komarnicki (2000) studied trace metal concentrations in tissues of the European mole (*Talpa europaea*) and calculated the annual increase rate in the concentration of cadmium in various organs. The highest rate was found in the kidney, reaching 12 mg/kg per year, while the annual increase in the liver concentration was 5 mg/kg. Millán et al. (2008) found higher Cd in the liver and muscles of adult foxes than in young individuals, and Perez-Lopez et al. (2015) noted a positive correlation of the liver and kidney Cd concentrations with the age of foxes*.* Lazarus et al. (2017) analysed a range of elements, including Cd, Hg, and Pb, in the livers, kidney, and muscles of a large number of individuals of top carnivores (brown bear, grey wolf, golden jackal, and Eurasian lynx) from Croatia. They noted that the age and sex were significant predictors of 24–35% of the total variability in Cd concentrations in the kidneys and livers of bears, and age explained 33–44% of the variability in Cd concentrations in all analysed wolf tissues.

Zinc is the physiologically crucial metal. Consequently, its concentration is strictly regulated by organisms and frequently shows no correlation with Zn concentration in the environment (Andrews et al. 1989). The Zn concentration was only elevated in the fox tissues in the area of highest contamination (the north-western part of Małopolska). While Zn concentrations in the organs of red foxes were elevated, they did not reach toxic levels given by Eisler (1993): 465 mg/kg d.w. in the liver and 274 mg/kg d.w. in the kidney. The median liver Zn concentration in red foxes from Małopolska, ranging from 119.2 to 146.0 μg/g d.w., was higher than in foxes from the Czech Republic (96.2–108 μg/g d.w.; Jankovska et al. 2010). The mean liver Zn concentration (130.6 μg/g d.w.) was similar to the concentration found in the livers of foxes from south-western Poland (128 μg/g d.w.; Binkowski et al.; 2016) and close to foxes from Switzerland (139.65 μg/g d.w; Dip et al. 2001—the value recalculated from the data on fresh mass basis, assuming 70% water content in soft tissues) and somewhat lower than in foxes from Hungary (156.93 μg/g d.w.; Heltai and Markov 2012). The lowest Zn liver concentration was noted in foxes from north-western Spain (77.0 μg/g d.w.; Perez-Lopez et al. 2015).

The median kidney Zn concentration found in the foxes from the Czech Republic (66.6–79.29 μg/g d.w.; Jankovska et al. 2010) was close to the median Zn concentration found in the kidney of foxes from Małopolska (69.39 to 77.96 μg/g d.w.). Similar to Zn concentration in the liver, the lowest kidney mean Zn concentration was found in foxes from north-western Spain (17.0 μg/g d.w.; Perez-Lopez et al. 2015), followed by foxes from south-western Poland (58.58 μg/g d.w.; Binkowski et al. 2016), Małopolska foxes (69.45–80.41 μg/g d.w.), Hungarian foxes (87.16 μg/g d.w.; Heltai and Markov 2012), and foxes from Switzerland (96.36 μg/g d.w.; Dip et al. 2001).

The mean Zn concentrations in muscles of Małopolska foxes ranged from 80.2 to 102. 24 μg/g d.w. and were close to those found in south-western Poland (87.31 μg/g d.w.; Binkowski et al. 2016).

The concentration of Zn in the liver of red foxes from the highly polluted area was similar in both sexes (142.3 in females vs. 144.93 in males). Also in other organs, the differences between the sexes were marginal: females had slightly higher Zn concentration in the kidney (F/M ratio = 1.2), while males had slightly higher Zn concentration in muscles (F/M ratio = 0.8).

When comparing Zn concentration in red fox tissues from different European countries, we noted its substantial variability in the livers (77.0 to 156.9 μg/g d.w.) and kidneys (17.0 to 96.36 μg/g d.w.). So, high variation in mean Zn tissue concentrations in different European populations of red foxes raises the question about the factors behind this phenomenon in case of the essential and well-regulated metal. In summary, our data showed that tissue level of Zn in foxes coming from a broad Małopolska pollution gradient is regulated clearly more efficiently than Cd and Pb, supporting the widely proven efficient regulation of Zn concentration in animal tissues.

The rodents can be considered one of the main sources of exposure of red foxes to toxic chemicals, particularly in agricultural areas (Heltai and Markov 2012). The comparison of metal concentrations in tissues of red foxes and the two common rodents showed that although Cd and Pb levels were higher in the red fox, the slope of the relationship between the concentrations of the two metals in the soil and the tissues of all species was the same. This means that it should be relatively easy to extrapolate the results obtained from wide-scale metal pollution monitoring programmes, performed on common rodents, to carnivores such as the red fox. Thanks to the common slope of the relationship between pollution level and metal concentrations in tissues, in the case of the red fox, this requires only applying metal-specific extrapolation factors (EF). Based on our study, we can derive such extrapolation factors for Cd and Pb. Comparing intercepts of the regressions describing the relationships of tissue metal concentration on soil metal concentrations, the EFs based on the bank vole are + 0.451 mg/kg for log_10_ Cd and + 0.368 mg/kg for log_10_ Pb, while those calculated for the yellow-necked mouse are + 0.523 mg/kg for log_10_ Cd and + 0.482 mg/kg for log_10_ Pb. However, because there were no significant differences in concentrations of these metals between the two rodent species, we can average the EFs for the two species, obtaining + 0.511 for mg/kg log_10_ Cd in the kidney and + 0.454 mg/kg for log_10_ Pb in the liver. These results are consistent with those reported by Markov et al. (2016), who compared the accumulation of trace metals in medium size carnivores, such as the golden jackal (*Canis aureus*) and the red fox, and in the rodent, fat dormouse (*Glis glis*), inhabiting agricultural environment in Bulgaria. They noted significantly higher Zn, Pb, and Cd concentrations in the liver and kidney of the carnivores than in the dormouse. However, the carnivores and the dormouse studied by Markov et al. (2016) came from different areas in Bulgaria, without a direct link between the trophic levels. Our Małopolska study is the first to our knowledge to investigate the accumulation of trace metals in the three mammalian species (a medium size carnivore and two rodents species) related directly by trophic relations and coming from the same areas with known concentrations of trace metals in soil. Our research brings a more complete and credible information on the transfer of trace metals from the soil to the higher trophic levels represented by wild species inhabiting a given area.

The important question is about the usefulness of the proposed extrapolation factors for other carnivorous species. Unfortunately, answering this question is impossible without case studies on at least a few other carnivorous species. Only having similar data sets for more species will allow us to see whether the rodent-to-carnivore EFs are more or less universal or are entirely species-specific. Some premises suggest that the former can be true, at least for certain groups of carnivores. For example, Reig and Jędrzejewski (1988) calculated the coefficients of food niche overlap (factor α, expressed as Pianka index, taking a value between 0 and 1) for predators from the Białowieża Forest, north-eastern Poland, in the winter and early spring. The diet of the red fox was compared with that of the pine marten (*Martes martes*), lynx (*Lynx lynx*), and a wolf (*Canis lupus*). The calculated α factors were 0.94, 0.68 and 0.57, respectively. The basis of the red fox diet is rodents, which account for about 84% of their diet in summer (Artois 1989; Goszczyński 1995). The composition of the diets of other small predators is also dominated by rodents; the main differences between the species are in the percentage of other prey in the diet. The diet of smaller predators had a larger share of amphibians and invertebrates (snails, insects, earthworms) (Jędrzejewska and Jędrzejewski 2001; Skalski and Wierzbowska 2008). It is also known that differences in the composition of the intestinal tract can cause very different effects of trace metal accumulation in species occurring in the same environment (Hamers et al. 2006; Smith et al. 2007). For example, the insectivorous common shrew (*Sorex araneus*) accumulates two to seven times more Cd and Pb in comparison with the herbivorous bank vole inhabiting the same area (Sawicka-Kapusta et al. 1994, 1999). The high similarity of diets and digestive systems in the red fox and other small carnivores allows assuming that extrapolation factors may have broader applicability than just between the two rodent species studied herein and the red fox.

On the other hand, thanks to the notable increase in the numbers of red fox populations in Poland and other European countries (Chautan et al. 2000), the wide geographic distribution of the species (Hersteinsson and Macdonald 1992), its tendency to colonise different types of environments (Goszczyński 1995), and relatively easy access to tissue samples through hunters, the red fox itself may become a good indicator of the impact of environmental pollution on carnivorous mammals, most of which are protected in Europe.

## Conclusions

I. Red foxes having their territories in the heavily polluted north-western part of the Małopolska province had substantially elevated Cd and Pb concentrations in their tissues, approaching the levels toxic to laboratory animals. However, the highest Pb concentrations were found in two individuals coming from urbanised areas of the otherwise relatively uncontaminated part of the province, indicating the importance of local contamination and diet specificity.
II. The comparison between the red fox and two rodent species originating from the same area revealed significantly higher concentrations of Pb and Cd in the livers and kidneys of the red fox. In all three species, concentrations of Pb and Cd depended on their concentrations in the soil. No such correlation was found for Zn.
III. The rate of increase of Pb concentration in the liver and Cd concentration in the kidney with increasing metal concentrations in the soil is the same for all three species. Thus, by using simple extrapolation factors (EF), the rodents can be used as indicators of metal contamination in red foxes and, possibly, also in other small carnivorous mammals. The estimated EFs are + 0.511 mg/kg for log_10_ Cd in the kidneys and + 0.454 mg/kg for log_10_ Pb in the livers.

## Electronic supplementary material

Fig. A1 The concentrations of Pb in different tissues (M, muscle; K, kidney; L, liver) of the red fox (*V. vulpes*); means ± 95% Tukey HSD intervals (PNG 147 kb)

High Resolution Image (TIF 10 kb)

Fig. A2 The concentrations of Pb in the tissues of females (F) and males (M) of the red fox (*V. vulpes*); means ± 95% Tukey HSD intervals (PNG 162 kb)

High Resolution Image (TIF 10 kb)

Fig. A3 The concentrations of Cd in different tissues (M, muscle; K, kidney; L, liver) of the red fox (*V. vulpes);* means ± 95% Tukey HSD intervals (PNG 21 kb)

High Resolution Image (EPS 998 kb)

Fig. A4 The concentrations of Cd in the tissues of females (F) and males (M) of the red fox (*V. vulpes*); means ± 95% Tukey HSD intervals (PNG 24 kb)

High Resolution Image (EPS 992 kb)

Fig. A5 The concentrations of Zn in different tissues (M, muscle; K, kidney; L, liver) of the red fox (*V. vulpes)*; means ± 95% Tukey HSD intervals (PNG 20 kb)

High Resolution Image (EPS 1004 kb)

Fig. A6 The concentrations of Pb in the livers of red fox (*V. vulpes*), yellow-necked mouse (*A. flavicollis*), and bank vole (*M. glareolus*); means with 95% Tukey HSD intervals (PNG 142 kb)

High Resolution Image (TIF 9 kb)

Fig. A7 Concentrations of Cd in the kidneys of the red fox (*V. vulpes*), yellow-necked mouse (*A. flavicollis*) and bank vole (*M. glareolus*); means and 95% Tukey HSD intervals. (PNG 21 kb)

High Resolution Image (EPS 1005 kb)
