# Supplementary figures and images for: Concentrations of cadmium and lead, but not zinc, are higher in red fox tissues than in rodents—pollution gradient study in the Małopolska province (Poland)
Source: Environ Sci Pollut Res Int. 2018 Dec 28;26(5):4961–74. doi: 10.1007/s11356-018-3951-5 (PMC6394479; doi:10.1007/s11356-018-3951-5)

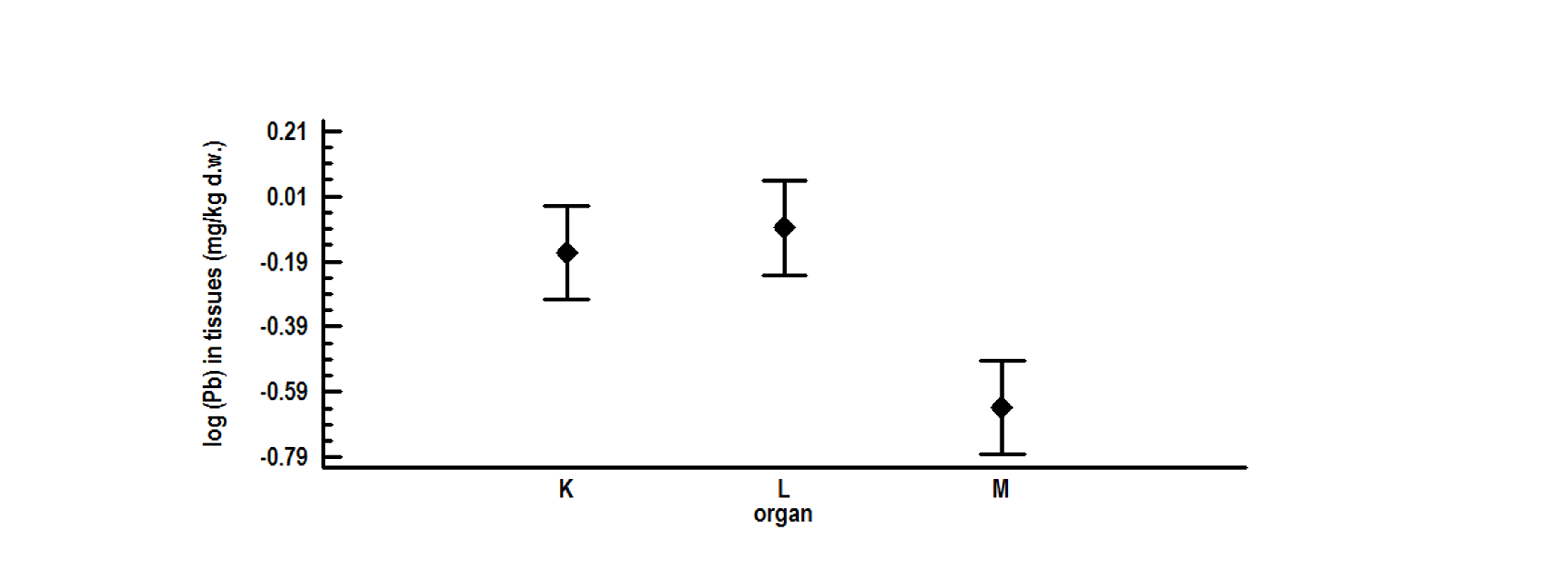

Supplement: Supplementary file 1 — The concentrations of Pb in different tissues (M, muscle; K, kidney; L, liver) of the red fox (V. vulpes); means ± 95% Tukey HSD intervals (PNG 147 kb) [file 11356_2018_3951_Fig5_ESM.png]

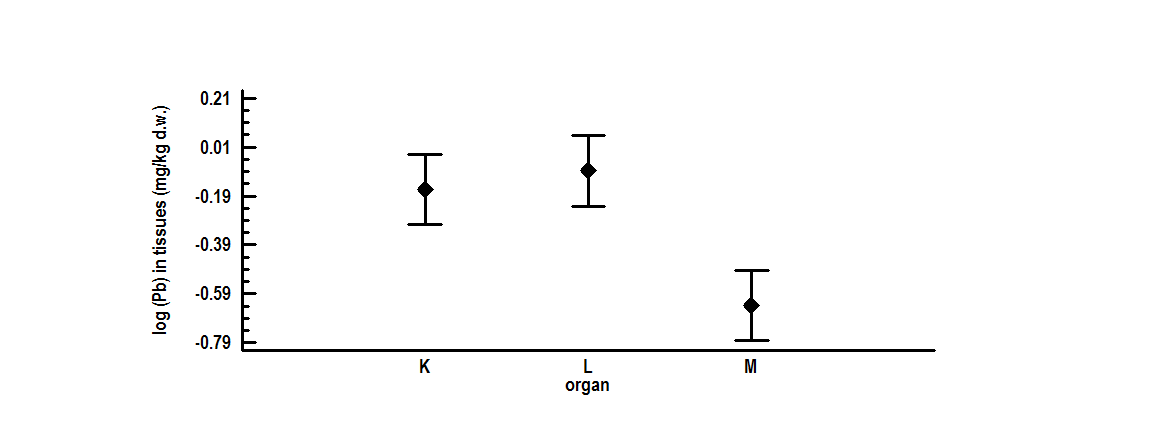

Supplement: Supplementary file 2 — High Resolution Image (TIF 10 kb) [file 11356_2018_3951_MOESM1_ESM.tif]

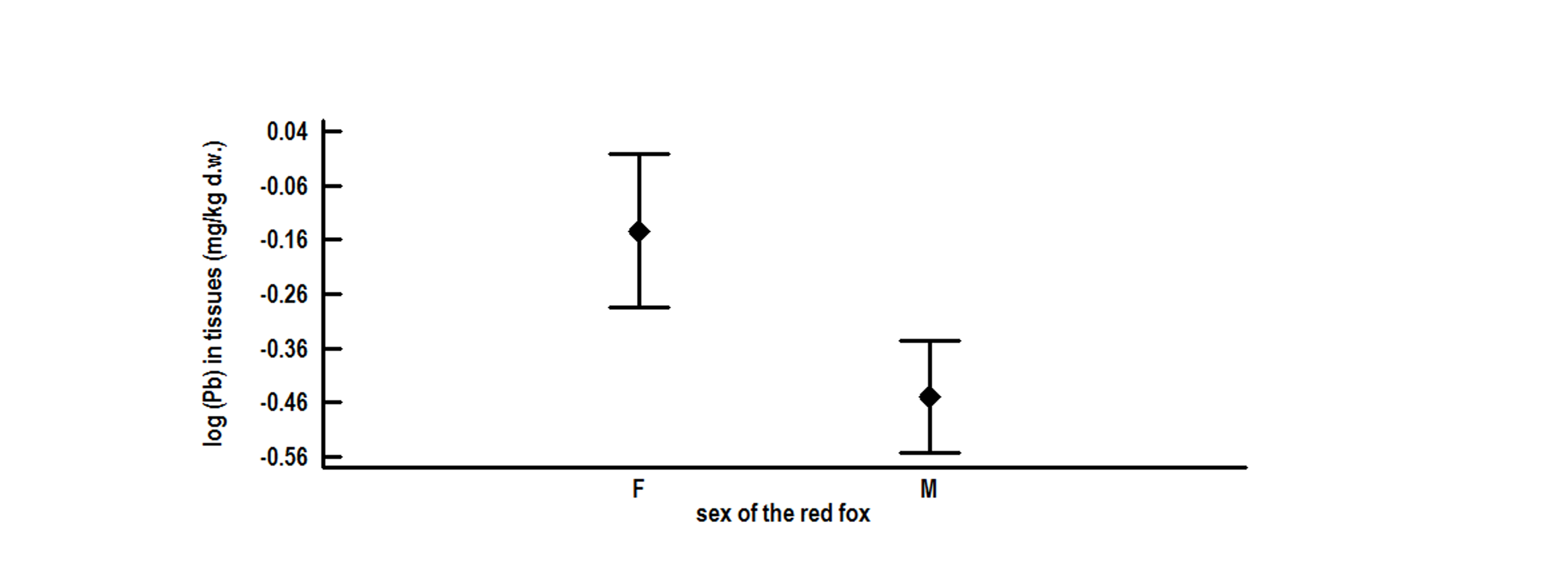

Supplement: Supplementary file 3 — The concentrations of Pb in the tissues of females (F) and males (M) of the red fox (V. vulpes); means ± 95% Tukey HSD intervals (PNG 162 kb) [file 11356_2018_3951_Fig6_ESM.png]

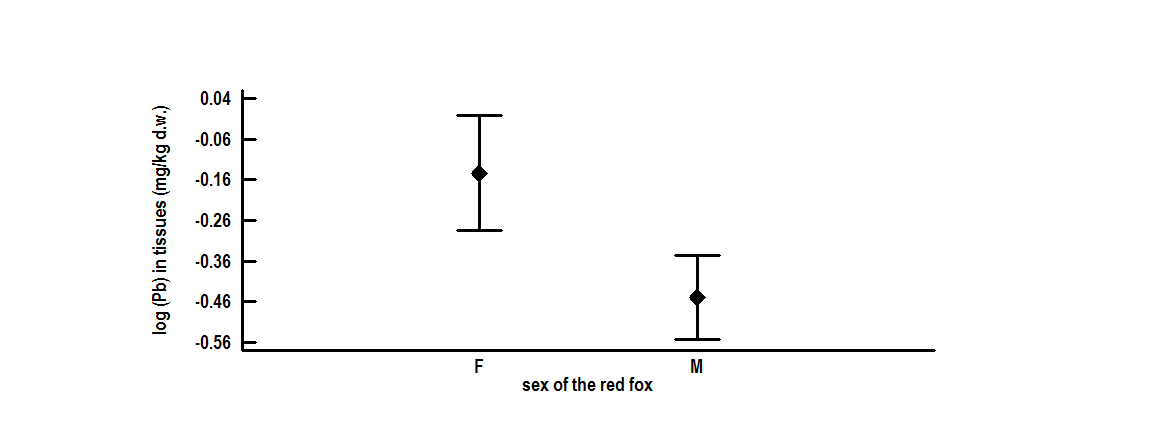

Supplement: Supplementary file 4 — High Resolution Image (TIF 10 kb) [file 11356_2018_3951_MOESM2_ESM.tif]

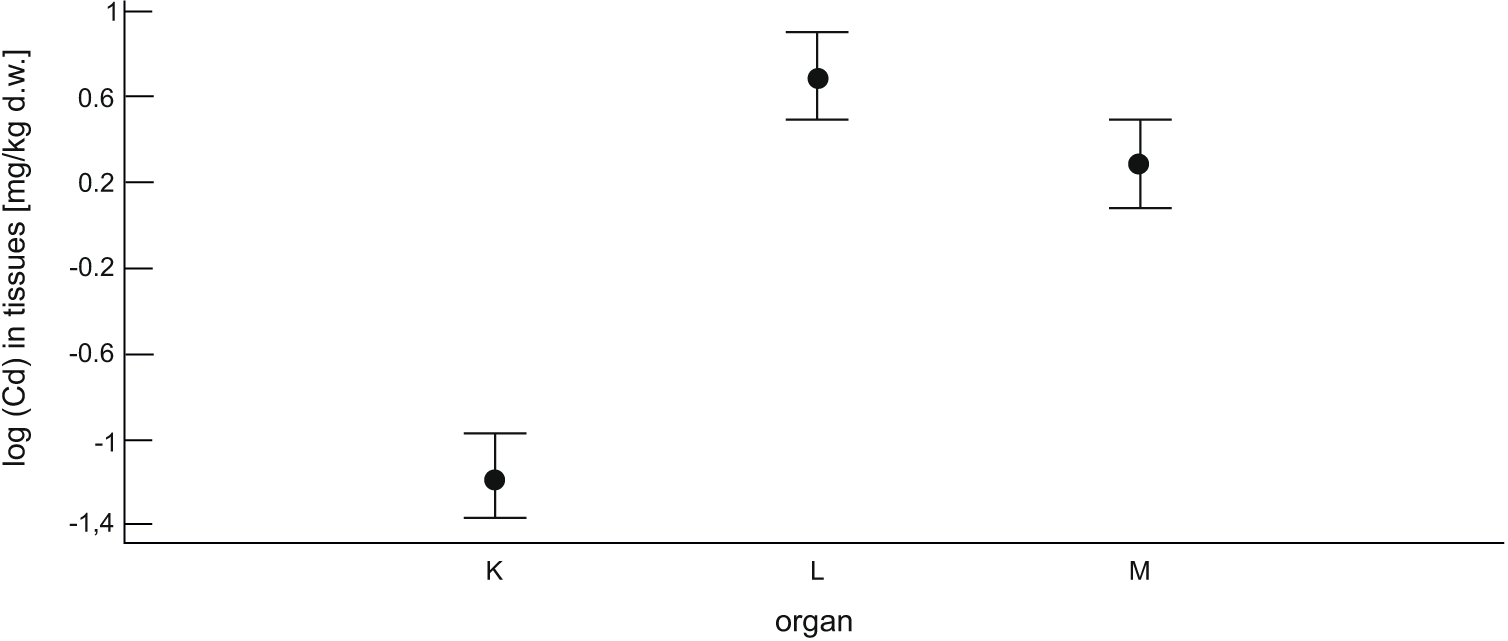

Supplement: Supplementary file 5 — The concentrations of Cd in different tissues (M, muscle; K, kidney; L, liver) of the red fox (V. vulpes); means ± 95% Tukey HSD intervals (PNG 21 kb) [file 11356_2018_3951_Fig7_ESM.png]

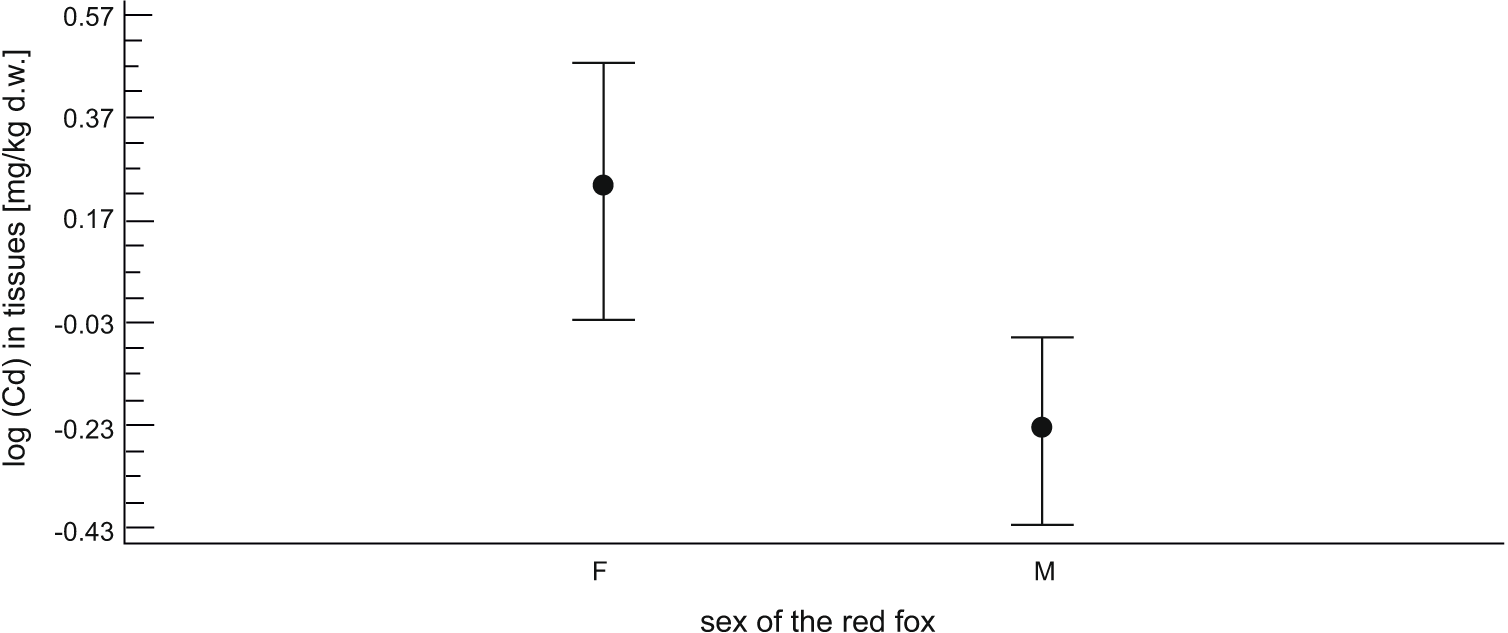

Supplement: Supplementary file 7 — The concentrations of Cd in the tissues of females (F) and males (M) of the red fox (V. vulpes); means ± 95% Tukey HSD intervals (PNG 24 kb) [file 11356_2018_3951_Fig8_ESM.png]

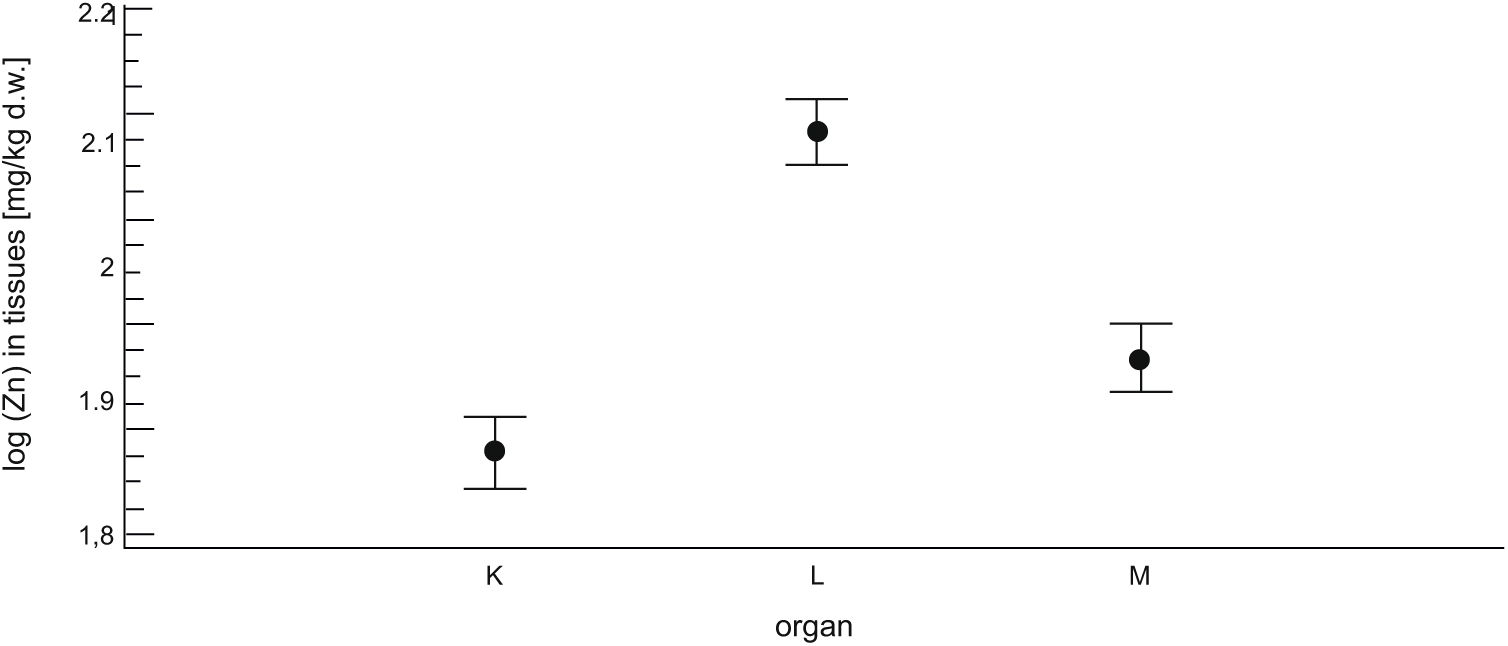

Supplement: Supplementary file 9 — The concentrations of Zn in different tissues (M, muscle; K, kidney; L, liver) of the red fox (V. vulpes); means ± 95% Tukey HSD intervals (PNG 20 kb) [file 11356_2018_3951_Fig9_ESM.png]

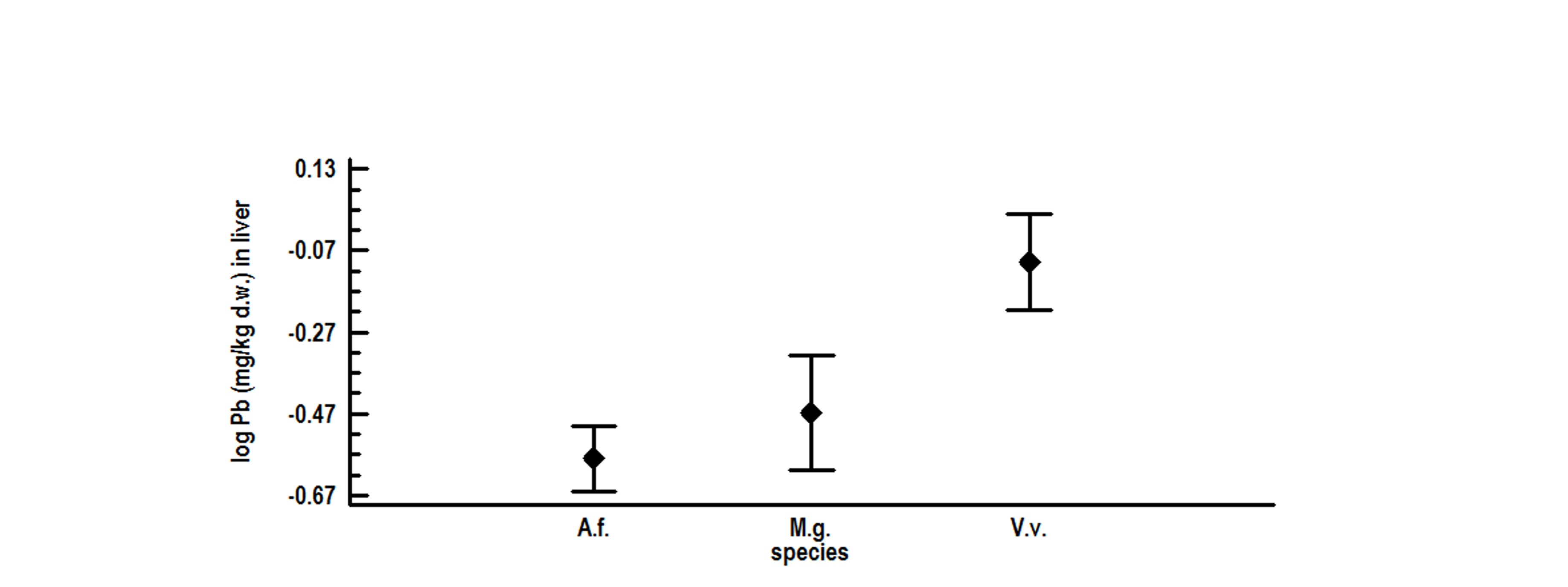

Supplement: Supplementary file 11 — The concentrations of Pb in the livers of red fox (V. vulpes), yellow-necked mouse (A. flavicollis), and bank vole (M. glareolus); means with 95% Tukey HSD intervals (PNG 142 kb) [file 11356_2018_3951_Fig10_ESM.png]

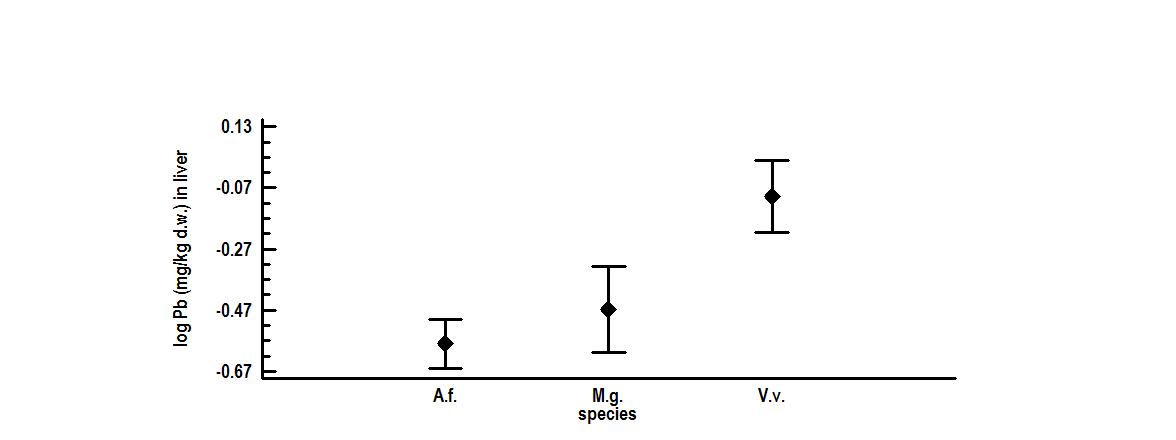

Supplement: Supplementary file 12 — High Resolution Image (TIF 9 kb) [file 11356_2018_3951_MOESM6_ESM.tif]

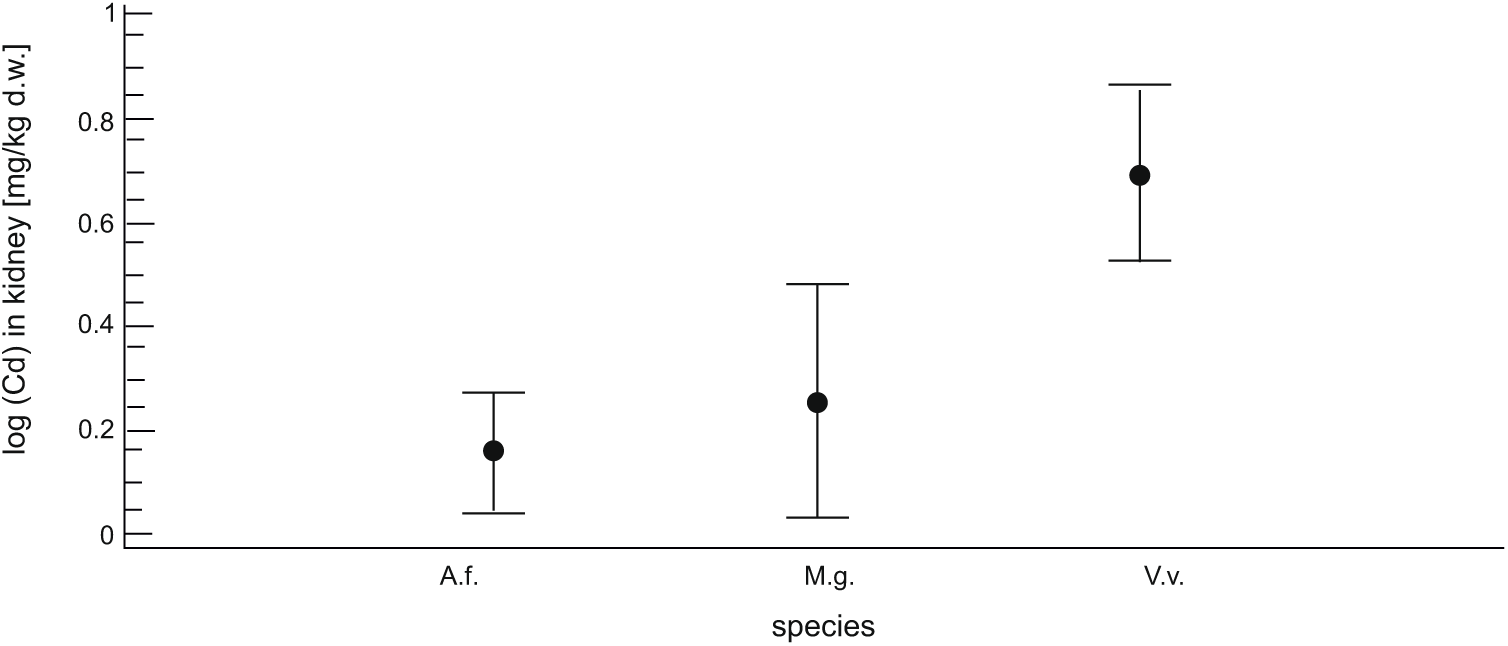

Supplement: Supplementary file 13 — Concentrations of Cd in the kidneys of the red fox (V. vulpes), yellow-necked mouse (A. flavicollis) and bank vole (M. glareolus); means and 95% Tukey HSD intervals. (PNG 21 kb) [file 11356_2018_3951_Fig11_ESM.png]
